# Selectivity of Exhaled Breath Biomarkers of Lung Cancer in Relation to Cancer of Other Localizations

**DOI:** 10.3390/ijms241713350

**Published:** 2023-08-28

**Authors:** Elina M. Gashimova, Azamat Z. Temerdashev, Dmitry V. Perunov, Vladimir A. Porkhanov, Igor S. Polyakov, Ekaterina V. Dmitrieva

**Affiliations:** 1 Department of Analytical Chemistry, Kuban State University, Stavropol’skaya St. 149, Krasnodar 350040, Russia; temerdashevaz@gmail.com (A.Z.T.); catherine_dmitrieva@outlook.com (E.V.D.); 2 Research Institute—Regional Clinical Hospital N° 1 n.a. Prof. S.V. Ochapovsky, 1 May St. 167, Krasnodar 350086, Russia; perunov007@rambler.ru (D.V.P.); kkb1@mail.ru (V.A.P.); i79282688844@gmail.com (I.S.P.)

**Keywords:** exhaled breath, volatile organic compounds, cancer biomarkers, GC-MS, diagnostics

## Abstract

Lung cancer is a leading cause of death worldwide, mostly due to diagnostics in the advanced stage. Therefore, the development of a quick, simple, and non-invasive diagnostic tool to identify cancer is essential. However, the creation of a reliable diagnostic tool is possible only in case of selectivity to other diseases, particularly, cancer of other localizations. This paper is devoted to the study of the variability of exhaled breath samples among patients with lung cancer and cancer of other localizations, such as esophageal, breast, colorectal, kidney, stomach, prostate, cervix, and skin. For this, gas chromatography-mass spectrometry (GC-MS) was used. Two classification models were built. The first model separated patients with lung cancer and cancer of other localizations. The second model classified patients with lung, esophageal, breast, colorectal, and kidney cancer. Mann–Whitney U tests and Kruskal–Wallis H tests were applied to identify differences in investigated groups. Discriminant analysis (DA), gradient-boosted decision trees (GBDT), and artificial neural networks (ANN) were applied to create the models. In the case of classifying lung cancer and cancer of other localizations, average sensitivity and specificity were 68% and 69%, respectively. However, the accuracy of classifying groups of patients with lung, esophageal, breast, colorectal, and kidney cancer was poor.

## 1. Introduction

Cancer is considered as one of the main problems of healthcare. A vast number of various forms and manifestations of cancer are widespread [1,2]. The most optimal cancer treatment outcome is in case of diagnostics in the early stage. The majority of cancers are prone to the lack of symptoms in the early stage, which leads to a high mortality rate due to late diagnostics. Therefore, the issue of developing new non-invasive techniques to serve cancer diagnostics is at hand.

Exhaled breath is being actively explored as a source of cancer biomarkers [3,4,5]. Owing to its simplicity and convenience of sampling as well as non-invasiveness, the interest in exhaled breath is gaining momentum. Various scientists published the results of studies where cancer diagnostic methods based on exhaled breath analysis using different analytical tools were developed [6,7,8,9]. Gas chromatography coupled with mass spectrometry (GC-MS) has taken a dominant position in the field of exhaled breath analysis since it is able to provide the most complete information regarding the sample composition [10,11,12]. Additionally, other analytical methods, including ion mobility spectrometry (IMS) [13], selected ion flow tube mass spectrometry (SIFT-MS) [14,15], proton-transfer-reaction mass spectrometry (PTR-MS) [16,17,18], are widely applied for exhaled breath analysis. Electronic noses (e-noses) can be considered as a separate group of tools for exhaled breath analysis with the advantages of simplicity of construction, mobility of the device, and high speed of analysis [19]. Various e-nose configurations are known to be good candidates as exhaled breath analysis instruments: an e-nose based on metal oxide semiconductor sensors [20,21], a chemoresistive e-nose [22], Cyranose 320 [23], aeonose [24,25], or combined devices consisting of several types of sensors [26,27]. Exhaled breath sampling techniques and analytical methods differ in the studies, which can influence the results. Alveolar, end-tidal, or mixed exhaled breath can be a subject of analysis. The concentration of endogenous VOCs is higher in samples of alveolar air [28]. However, the sampling of alveolar air involves using sophisticated equipment, which restricts the mobility and velocity of sampling. Sampling of end-tidal exhaled air allows us to take more alveolar air, but the ratio of alveolar and dead space air in a sample may differ from one person to another, which contributes to a distortion of the results. Mixed exhaled air is highly diluted by dead space air; therefore, the number of endogenous VOCs is lower. However, this approach is simple, quick, and does not require sophisticated equipment. Obtaining reliable results using mixed exhaled air is possible only in the case of the strict controlling of ambient air as well as conducting the sampling procedure [29].

Attempts to create a diagnostic method using exhaled breath to reveal cancer of various localizations have already been demonstrated [30,31]. The majority of studies are devoted to the identification of an exact disease, as a rule, lung [21,24,26,27] or breast [6,23] cancer. Benzene, 2-propanol, styrene, and pentane were often assigned as metabolites linked with lung cancer development [32]. Breast cancer biomarkers in common significantly differ in various studies [23,33]. However, heptanal was noted as a biomarker in several studies [34,35]. Exhaled breath can be useful for diagnosing cancer of other localizations. For example, a diagnostic model was created in [36], which allowed for the identification of cirrhosis, and primary and secondary liver tumors. Ethane, (E)-2-nonene, acetaldehyde, and acetone contributed the most to the diagnostic accuracy. Ovarian cancer can be diagnosed with 89% accuracy using a diagnostic model based on decanal, nonanal, styrene, 2-butanone, and hexadecane, which were identified in exhaled breath using GC-MS [37]. Cyclohexanone, 2,2-dimethyldecane, dodecane, 4-ethyl-1-octyn-3-ol, ethylaniline, cyclooctylmethanol, trans-2-dodecen-1-ol, 3-hydroxy-2,4,4-trimethylpentyl, 2-methylpropanoate, and 6-t-butyl-2,2,9,9-tetramethyl-3,5-decadien-7-yne were assigned to colorectal cancer biomarkers [38].

Another interesting issue is to find alternative evidence that the tumor affects VOC levels in exhaled breath. It can be achieved by comparing exhaled breath profiles of patients before and after surgery. This approach was demonstrated on 84 patients with lung cancer [39]. Concentrations of 2,5-dimethylfurane, cyclohexanone, propyl cyclohexane, octanal, nonanal, decanal, and 2,2-dymethyldecane differed the most in exhaled breath of patients with lung cancer before and after surgery. An alternative approach is to study the VOC profile extracted by cancer cell lines. The authors [40] compared metabolite profiles of adenocarcinoma, squamous cell carcinoma, large cell carcinoma, small cell carcinoma cell lines, and one normal small airway epithelial cells. Benzaldehyde, 2-ethylhexanol, and 2,4-decadien-1-ol were found as potential lung cancer biomarkers. Comparing profiles of VOCs from different subjects allows one to trace metabolic pathways and obtain additional proof of biomarkers’ origins. Correlations between the results of exhaled breath and fecal samples of patients with gastric cancer were found in study [41].

Considering the highest mortality rate and sophisticated diagnostic procedures applied in clinical practice nowadays, the development of a non-invasive and accurate lung cancer diagnostic tool is the most urgent task [2,8,42]. A conventional approach to developing a diagnostic method is the comparison of healthy volunteers and patients with the studied disease. However, the accuracy of the diagnostic model can be unknown when it comes to other diseases. Therefore, it is essential to consider the accuracy of biomarkers not only in relation to heathy subjects, but the selectivity of potential biomarkers to other diseases. Some studies have considered the development of a diagnostic method able to simultaneously detect several cancer types, for example, an electronic nose was presented in [30] consisting of an array of cross-reactive nanosensors based on organically functionalized gold nanoparticles for diagnosing lung, breast, colorectal, and prostate cancer. VOC profiles of patients with lung cancer, lung cancer and COPD, COPD, and healthy subjects were compared in the study [42].

The paper is focused on the selectivity of exhaled breath analysis using GC-MS to distinguish lung cancer from cancer of other localizations. Breast, esophageal, colorectal, kidney, prostate, cervix, and skin cancer localizations were considered.

## 2. Results

The study includes two groups of cancer patients: 85 patients with lung cancer and 85 patients with cancer of other organs, including 11 patients with esophageal cancer, 22 patients with mammary cancer, 16 patients with colorectal cancer, 14 patients with kidney cancer, 7 patients with stomach cancer, 6 patients with prostate cancer, 5 patients with cervix cancer, and 4 patients with skin cancer. These samples of exhaled breath were analyzed using GC-MS.

VOCs and their ratios, which were different in lung cancer and other cancer localization groups, were found using a Mann–Whitney U test. Hexane (*p* = 0.013), acetonitrile (*p* = 0.036), 1-methylthiopropene (*p* = 0.010), 1-methylthiopropane (*p* = 0.006), and dimethyl sulfide (*p* = 0.021) show a significant difference between groups of patients with lung cancer and cancer of other localizations. Also, several ratios were significantly different between lung cancer and cancer of other localizations (Table 1).

The ratios were used as input values for the creation and validation of the diagnostic model using an artificial neural network (ANN). The accuracy for training, validation, and test datasets was calculated. The efficiency of Broyden–Fletcher–Goldfarb–Shanno (BFGS) and nonlinear conjugate gradient algorithms was compared for the creation of the model. To validate the model, three-fold cross-validation was implemented (Table 2). As seen from Table 2, the BFGS algorithm is better on a test dataset.

The variability of exhaled breath samples of patients with lung, esophageal, breast, colorectal, and kidney cancer was estimated by Kruskal–Wallis H tests. Each pairwise comparison was conducted using Mann-Whitney U tests with a subsequent adjustment of *p*-value for false discovery rate (FDR).

**Figure 1:**
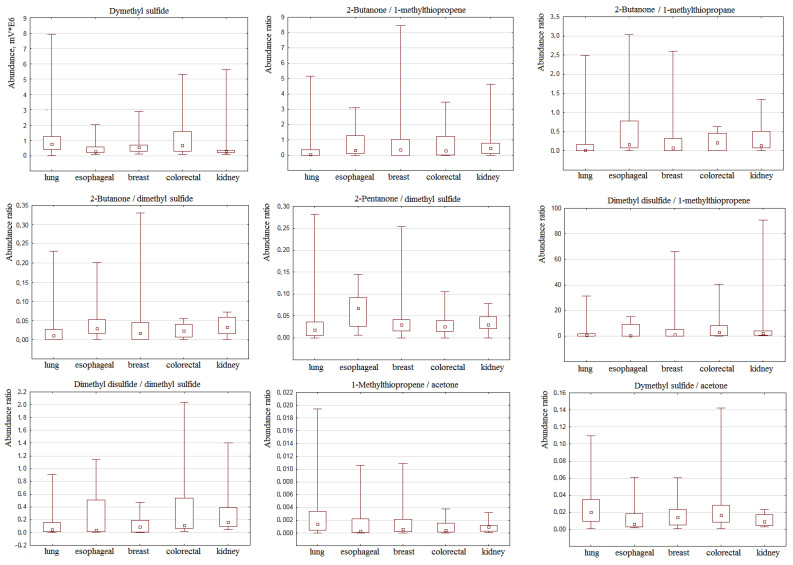
Median and interquartile range of peak areas and their ratios with the highest difference between groups of lung, esophageal, breast, colorectal, and kidney cancer.

Some VOCs were found to be different in the studied groups (Table 3). Figure 1 represents a median and interquartile range of parameters with the lowest *p*-value.

Discriminant analysis (DA) was applied to classify groups of patients with lung, esophageal, breast, colorectal, and kidney cancer. Ratios of VOCs, which were significantly different between the groups, were used as input values. The DA classification matrix is presented in Table 4.

**Figure 2:**
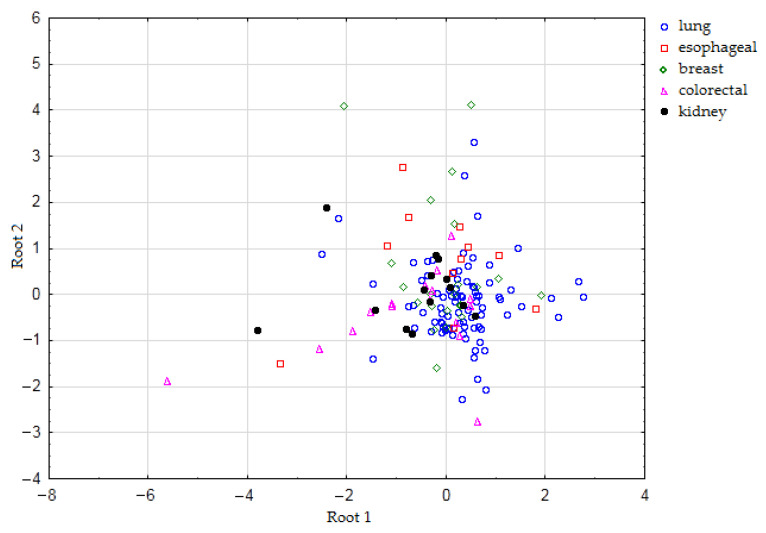
Scattering diagram of canonical values for exhaled breath samples depending on cancer localization.

Figure 2 represents a scattering diagram of canonical values for exhaled breath samples depending on cancer localization.

In addition, the gradient-boosted decision trees (GBDT) algorithm was used to separate groups of patients with lung, esophageal, breast, colorectal, and kidney cancer. To validate the model, three-fold cross-validation was used. The performance for training and test datasets was calculated (Table 5).

## 3. Discussion

The development of a non-invasive cancer diagnostic method is an urgent challenge, which attracts the attention of many researchers worldwide [4,8,10,18,21]. Despite the attempts of many research groups to solve the problem, the breath test for cancer diagnostics has not yet been implemented in clinical practice. It can be explained by the many pitfalls that are often omitted during research. A conventional approach of biomarker identification assumes comparing a group of pathology with a group of healthy volunteers. However, the approach can lead to false-positive results linked to a lack of considering other disorders. An issue of this work was to compare groups of patients with cancer of various localizations. Breast, esophageal, colorectal, kidney, prostate, cervix, and skin cancers were considered. Not only peak areas but also their ratios were considered in terms of the difference between lung cancer and cancer of other localizations. The implementation of this approach was demonstrated earlier [43].

Taking into account difficulties concerning lung cancer diagnostics, the most essential task was to separate samples of exhaled breath of lung cancer patients and patients with cancer of other localizations. For this, a Mann–Whitney U test was applied. Acetonitrile, 1-methylthiopropene, 1-methylthiopropane, and dimethyl sulfide were different between patients with lung cancer and cancer of other localizations. Acetonitrile [44], dimethyl sulfide [45], and 1-methylthiopropene [46] were determined as lung cancer biomarkers earlier. Dimethyl sulfide was also listed as a putative biomarker of esophageal cancer [17]. The ratio of 1-methylthiopropane/acetone was different in groups of lung cancer and healthy volunteers in the previous work [43].

To create a model capable of separating patients with lung cancer and patients with other cancer localizations, ANN was used. ANN is one of the most powerful machine-learning algorithms. It was used in many research works to create diagnostic models [24,47]. Our previous research has shown that the diagnostic model created using ANN is more accurate than random forest, support vector machine, and logistic regression on the same dataset [43]. ANN is the most flexible method capable of revealing complex patterns that may be inaccessible to traditional algorithms. Therefore, ANN was used in this work to create a classification model to separate lung cancer patients from patients with cancer of other localizations. The efficiency of two algorithms: Broyden–Fletcher–Goldfarb–Shanno (BFGH) and nonlinear conjugate gradient was compared to train the ANN. The nonlinear conjugate gradient algorithm is attractive due to the simplicity of the iterations and lower storage requirements [48]. BFGS is one of the most effective quasi-Newton methods [49]. BFGS surpassed the conjugate gradient algorithm: the average sensitivity and specificity on the test dataset were 67% and 69% for BFGS and 56% and 57% for conjugate gradient. Accuracy, which is achieved by comparing lung cancer patients with healthy individuals, is significantly greater in most cases [50,51,52,53]. The accuracy obtained in our research is utterly inadequate for a large-scale screening due to the high number of expected false positives. The study has several limitations: the group of patients with other cancer localization includes uneven distribution of various cancer localizations. Another drawback is the sample size, which is too small to obtain reliable results. However, this study highlights the problem of differentiating various diseases through exhaled breath analysis. Prospectively, the diagnostic models aimed to identify lung cancer may classify patients with cancer of various localizations as lung cancer patients. Therefore, it is essential to compare not only samples of lung cancer patients and healthy volunteers but also consider other pathologies, which can be potentially confused with the disease.

Another task of this work was to evaluate the possibility of classifying patients with various cancer localizations, namely lung, esophageal, breast, colorectal, and kidney cancer, and find the parameters specific to each group. For this, a Kruskal–Wallis H test was used. As can be seen from Figure 2, there are no parameters that can classify each cancer in the separate groups. However, the level of dimethyl sulfide is elevated in the case of lung and esophageal cancer in comparison with other cancer localizations. The majority of ratios containing sulfuric compounds is higher in the case of esophageal and colorectal cancers. Dimethyl sulfide and ratios containing this component were significantly different in groups of lung and esophageal cancer as well as lung and kidney cancer. Levels of the set of VOCs and their ratios were equal for the rest of the cancer localizations (Table 2).

An attempt to classify lung, esophageal, breast, colorectal, and kidney cancer using DA was applied owing to the ability of visualization using a scattering diagram of canonical values. As shown in Figure 2, the exhaled breath samples of patients with cancer of various localizations cannot be separated. Most samples of esophageal, breast, colorectal, and kidney cancer are classified as lung cancer. ANN is one of the most effective machine learning algorithms [38]. It is worth noting that ANN works better when the groups have an equal number of cases. Considering the task of separation of groups with different numbers of observations, one of the most effective machine-learning algorithms is GBDT [45], which was applied to classify the exhaled breath samples of patients with cancer of different localizations. The accuracy of classification on the training data was relatively high for lung and esophageal cancer, but on the test data, it was significantly worse for all cancer localizations. Among the studied cancer types, the model better recognized lung and breast cancer on the test dataset (Table 5). Lung, breast, colorectal, and prostate cancers were classified through exhaled breath analysis using electronic nose based on cross-reactive nanosensors [30]. The groups of patients with lung, breast, and colon cancer were fully separated, but prostate and lung cancer and healthy individual groups were overlapped. Our study also demonstrates a better separation of lung and breast cancer, but accuracy is significantly lower. The main limitation of this part of the study is a small sample size with a lot of comparable groups, each of which contains a low number of samples.

The exhaled breath VOC profiles of lung cancer patients and patients suffering from other lung diseases (e.g., chronic obstructive pulmonary disease (COPD), asthma, pneumonia, pulmonary embolism, benign lung tumors) as well as healthy controls were compared in this study [42]. It was shown that the discrimination of lung cancer and healthy controls was better than between lung cancer and other lung diseases. The classification of 50 breast cancer patients, COPD patients, and healthy volunteers was fulfilled with 100% accuracy on test data using hemoresistive gas sensors and canonical analysis of principal coordinates [54].

The results obtained in this study additionally prove the assumption of obtaining a potentially incorrect diagnosis since the samples of patients with cancer of various localizations are poorly separated. The issue of separating cancer of various localizations is essential for the development of a reliable and accurate cancer diagnostic tool.

## 4. Materials and Methods

### 4.1. Materials

Acetone and 1-propanol (99.9%) were obtained from Ecos-1 (Moscow, Russia); *o*-xylene, *p*-xylene, *m*-xylene, and 2-propanol (>95%) were obtained from Vecton (Moscow, Russia); acetonitrile, ethanol, benzene, n-hexane, methanol, toluene (>95%) and octane, nonane, decane, and dodecane (≥99%) were purchased from Sigma-Aldrich (St. Louis, MO, USA).

### 4.2. Study Participants

The study includes 2 groups of cancer patients: 85 patients with lung cancer and 85 patients with cancer of other organs, including 11 patients with esophageal cancer, 22 patients with mammary cancer, 16 patients with colorectal cancer, 14 patients with kidney cancer, 7 patients with stomach cancer, 6 patients with prostate cancer, 5 patients with cervix cancer, and 4 patients with skin cancer. All patients involved in the study were examined in the State budgetary healthcare institution “Research Institute—Regional Clinical Hospital N° 1 named after Professor S.V. Ochapovsky”. Biopsy results were used for diagnosis confirmation. The samples were collected at the stage of the diagnosis verification before treatment. The data on the participants are summarized in Table 6.

### 4.3. Exhaled Breath Collection and GC-MS Analysis

Considering the simplicity and mobility of mixed expiratory breath sampling, this approach was chosen to collect the samples. Mixed expiratory breath was collected in 5 L Tedlar (Supelco, Bellefonte, PA, USA) sampling bags. Nitrogen was used for cleaning the bags. The participants provided the samples of exhaled breath in the hospital. Ambient air was used as a blank sample. The patients underwent overnight fasting before sampling. Smokers were not involved in the study if they smoked less than 2.5 h prior to breath collection. Exhaled breath was sampled after a 10 min rest of the participant in a separate sampling room. Patients were asked to breathe, hold their breath for 10 s, and breathe out into the sampling bag. The procedure was repeated until the sampling bag was filled. Sample treatment and chromatographic analysis conditions were optimized and applied earlier [43,55]. A PV-2 aspirator (Chromatec, Yoshkar-Ola, Russia) and Tenax TA (60–80 mesh, Chromatec, Yoshkar-Ola, Russia) sorbent tubes were applied to preconcentrate the samples. The rate and time of aspiration were 200 mL/min and 2.5 min, respectively. A system consisted of a gas chromatograph (Chromatec crystal 5000.2, Chromatec, Yoshkar-Ola, Russia) combined with a quadrupole mass spectrometer with an electron ionization source (Chromatec MSD, Chromatec, Yoshkar-Ola, Russia) and a two-stage thermal desorber TD2 (Chromatec, Yoshkar-Ola, Russia). Separation of analytes was conducted using a Supelco Supel-Q PLOT (30 m × 0.32 mm × 15 μm) column (Bellefonte, PA, USA). Data were acquired using Chromatec Analytic (Chromatec, Yoshkar-Ola, Russia) software and mass spectral library NIST 2017, Version 2.3 (Gatesburg, PA, USA). GC-MS analysis conditions are presented in Table 7.

### 4.4. Statistical Analysis

The chromatograms of exhaled breath samples were obtained in a full scan mode. Peak areas of individual VOCs were calculated in extracted ion chromatogram (EIC) mode. The influence of ambient air was eliminated by subtraction of room air peak area values from the exhaled breath ones. Negative results of subtraction were set to zero. VOCs with peak area values exceeding room air levels by at least 50% were selected for statistical analysis. Another criterion for selection was occurring the VOC in more than 50% of samples. The ratios of the compound peak areas to the main ones (present in more than 88% of the samples) as well as ratios of the main VOCs were considered for statistical analysis. The description of ratio calculations was presented earlier in detail [43].

StatSoft STATISTICA (version 10) was applied for statistical analysis and building the diagnostic model. At the first stage, the normality of data distribution was checked applying Kolmogorov–Smirnov test. The distribution was not normal. Therefore, a Mann–Whitney U test (*p* = 0.05) was used to select the VOCs and their ratios, which are different in groups of lung cancer patients and patients with cancer of other organs. A Kruskal–Wallis H test (*p* = 0.05) was used to assess the mean differences between groups of patients with lung, esophageal, breast, colorectal, and kidney cancer. Each pairwise comparison was conducted using a Mann–Whitney U test. A Type I error from multiple hypothesis testing was addressed with False Discovery Rate (FDR) correction using the Benjamini–Hochberg adjusted *p*-value cutoff of ≤0.05.

The diagnostic model for selecting lung cancer patients from patients with other cancer localizations was built by applying a multilayer perceptron artificial neural network (ANN). A recurrent feedforward ANN with fully connected one hidden layer was created. Model training was fulfilled using 2 algorithms: Broyden–Fletcher–Goldfarb–Shanno and nonlinear conjugate gradient. The model has the following structure: the input layer consists of 15 neurons corresponding to VOC, which were significantly different between the studied groups (Table 1), a hidden layer, including 7 neurons, and the output layer, including 2 neurons, which corresponded to the group of lung cancer or cancer of other organs. The hidden layer activation function was identity, output layer—SoftMax. The data were divided into 3 datasets: training (60%), validation (10%), and test (30%). To provide reliable results, three-fold cross-validation was used.

To create the model allowing classification of patients with different cancer localizations, the gradient-boosted decision trees (GBDT) algorithm was applied. The data were divided into 2 datasets: training (70%) and test (30%). The reliability of the results was provided by three-fold cross-validation.

## 5. Conclusions

In the present research, we shed a light on the problem of classifying patients with various diseases using exhaled breath analysis. The classical approach supposes the comparison of patients with certain diseases with healthy persons, but putative biomarkers can indicate not only the investigated diseases but other pathologies. To avoid incorrect classification, the other pathologies must be considered before the implementation of exhaled breath tests in clinical practice. Exhaled breath VOC profiles of patients with cancer of various localizations were considered in this work. The results obtained prove the assumption about overlapping the VOC profiles of patients with various cancer localizations. Further research is required to determine biomarkers specific to each cancer localization type for providing accurate diagnosis.

## Figures and Tables

**Table 1:** VOCs and their ratios, which have a significant difference between groups of patients with lung cancer and cancer of other localizations.

VOC or VOC Ratio	*p*-Value
Acetonitrile	0.035681
1-Methylthiopropene	0.010414
1-Methylthiopropane	0.005953
Dimethyl sulfide	0.021984
2-Butanone/1-methylthiopropene	0.000567
2-Butanone/1-methylthiopropane	0.002528
2-Butanone/dimethyl sulfide	0.004545
Allyl methyl sulfide/dimethyl disulfide	0.033031
Allyl methyl sulfide/acetone	0.045749
2-Pentanone/1-methylthiopropene	0.008109
2-Pentanone/1-methylthiopropane	0.024048
2-Pentanone/dimethyl sulfide	0.030791
Dimethyl disulfide/1-methylthiopropene	0.044414
Dimethyl disulfide/dimethyl sulfide	0.031523
1-Methylthiopropene/isoprene	0.047466
1-Methylthiopropene/acetone	0.012438
1-Methylthiopropane/isoprene	0.041529
1-Methylthiopropane/acetone	0.025859
Dimethyl sulfide/acetone	0.049961

**Table 2:** Performance of ANN models.

Algorithm	Dataset	Training Dataset		Validation Dataset		Test Dataset	
		Sensitivity	Specificity	Sensitivity	Specificity	Sensitivity	Specificity
Broyden–Fletcher–Goldfarb–Shanno	1	62	62	89	60	66	68
	2	77	56	70	100	57	69
	3	80	87	70	50	79	71
Nonlinear conjugate gradient	1	66	63	89	80	55	43
	2	81	48	70	100	71	59
	3	43	77	30	75	41	79

**Table 3:** Statistically significantly different parameters in groups of patients with lung, esophageal, breast, colorectal, and kidney cancer (bold *p*-values were statistically significant).

VOC or VOC Ratio	Kruskal–Wallis H Test *p*-Value	Mann–Whitney U Test *p*-Value									
		Lung vs. Esophageal	Lung vs. Breast	Lung vs. Colorectal	Lung vs. Kidney	Esophageal vs. Breast	Esophageal vs. Colorectal	Esophageal vs. Kidney	Breast vs. Colorectal	Breast vs. Kidney	Colorectal vs. Kidney
Dimethyl sulfide	**0.0056**	**0.0076**	0.0743	0.8634	**0.0040**	0.1752	0.1323	0.9782	0.4688	0.1313	0.1095
2-butanone/1-methylthiopropene	**0.0037**	0.0143	0.0480	0.0403	0.0058	0.5929	0.6044	0.8695	0.9882	0.6496	0.7870
2-butanone/1-methylthiopropane	**0.0093**	0.0089	0.4593	0.1086	0.0101	0.0929	0.1748	0.4434	0.3517	0.2299	0.4669
2-butanone/dimethyl sulfide	**0.0057**	0.0124	0.1849	0.0683	0.0054	0.2519	0.3001	1.0000	0.8360	0.2299	0.1834
2-pentanone/dimethyl sulfide	**0.0095**	**0.0019**	0.0569	0.3265	0.1184	0.1314	0.0318	0.0846	0.5059	0.9353	0.5194
Dimethyl sulfide/acetone	**0.0057**	**0.0079**	0.0743	0.3948	**0.0049**	0.2599	0.0983	0.4273	0.4688	0.3223	0.1190
Dimethyl disulfide/dimethyl sulfide	**0.0077**	0.6620	0.7490	0.0158	**0.0014**	0.6331	0.2462	0.0846	0.0891	0.0363	0.5194
1-methylthiopropene/acetone	**0.0321**	0.0674	0.0671	0.0179	0.1115	0.6468	0.9410	0.3380	0.5643	0.8584	0.4928
Dimethyl disulfide/1-methylthiopropene	**0.0272**	0.9358	0.3035	0.0219	0.0057	0.7168	0.2887	0.0589	0.3994	0.2237	0.9503

**Table 4:** Classification matrix of DA model.

Cancer	Sample Classification Results						
	Lung	Esophageal	Breast	Colorectal	Kidney	Total	Percentage of Correct Classification, %
**Lung**	81	3	1	0	0	85	95
**Esophageal**	9	1	0	1	0	11	9
**Breast**	18	2	2	0	0	22	9
**Colorectal**	12	0	0	4	0	16	25
**Kidney**	12	0	0	1	1	14	7

**Table 5:** Classification matrix of GBDT model.

Sample Classification Results									
Data	Cancer	Dataset	Lung	Esophageal	Breast	Colorectal	Kidney	Total	Percentage of Correct Classification, %
**Training**	**Lung**	1	46	3	1	6	0	56	82
	**Esophageal**		3	4	0	0	1	8	50
	**Breast**		6	1	2	5	0	14	14
	**Colorectal**		3	0	1	7	0	11	64
	**Kidney**		4	2	1	1	1	9	11
**Test**	**Lung**		20	3	1	5	0	29	69
	**Esophageal**		1	2	0	0	0	3	67
	**Breast**		5	2	0	1	0	8	0
	**Colorectal**		3	1	0	1	0	5	20
	**Kidney**		2	0	0	3	0	5	0
**Training**	**Lung**	2	40	7	5	2	3	57	70
	**Esophageal**		2	2	0	0	3	7	29
	**Breast**		4	3	6	0	2	15	40
	**Colorectal**		4	2	1	1	2	10	10
	**Kidney**		4	0	4	0	2	10	10
**Test**	**Lung**		16	5	6	0	1	28	57
	**Esophageal**		2	0	0	0	2	4	0
	**Breast**		3	1	3	0	0	7	43
	**Colorectal**		3	0	3	0	0	6	0
	**Kidney**		3	0	0	0	1	4	25
**Training**	**Lung**	3	46	1	5	2	2	56	82
	**Esophageal**		0	7	0	0	0	7	100
	**Breast**		0	0	15	0	0	15	100
	**Colorectal**		0	0	0	11	0	11	100
	**Kidney**		0	0	0	0	9	9	100
**Test**	**Lung**		18	0	6	3	2	29	62
	**Esophageal**		1	0	2	1	0	4	0
	**Breast**		2	0	4	0	1	7	57
	**Colorectal**		1	0	2	0	2	5	0
	**Kidney**		2	1	2	0	0	5	0

**Table 6:** Information on the participants.

Group of Patients	Number	Male/Female	Age (Median, Range)	Smokers
Lung cancer	85	63/22	66, 30–79	32
Esophageal cancer	11	10/1	61, 45–74	5
Breast cancer	22	22	60, 30–73	1
Colorectal cancer	16	6/10	66, 35–85	1
Kidney cancer	14	8/6	63, 49–81	6
Stomach cancer	7	5/2	64, 54–79	2
Prostate cancer	6	6	69, 58–76	2
Cervix cancer	4	4	57, 42–61	1
Skin cancer	4	2/2	64, 59–67	1

**Table 7:** GC-MS analysis conditions.

Equipment	Parameter	Value
Thermal desorber	Carrier gas	Helium
	Carries gas flow rate (desorption from the sorption tube), mL/min	30
	Desorption temperature, °C	250
	Initial trap temperature, °C	−10
	Final trap temperature, °C	250
	Carrier gas flow rate (desorption from the trap), mL/min	50
	Desorption time, min	5
	Speed of the trap heating, °C/min	2000
GC-MS	Carrier gas	Helium
	Injector temperature, °C	250
	Split ratio	1:10
	Ion source temperature, °C	200
	Transfer line temperature, °C	250
	Scan range, amu	29–250
	Electron impact ionization, eV	70

## Data Availability

Not applicable.
